# Escitalopram in the prevention of posttraumatic stress disorder: a pilot randomized controlled trial

**DOI:** 10.1186/s12888-015-0391-3

**Published:** 2015-02-19

**Authors:** Sharain Suliman, Soraya Seedat, Janine Pingo, Taryn Sutherland, Joseph Zohar, Dan J Stein

**Affiliations:** MRC Anxiety Disorders Unit, Department of Psychiatry, Stellenbosch University, Cape Town, South Africa; Department of Psychiatry, Tel-Aviv University, Tel-Aviv, Israel; MRC Anxiety Disorders Unit and Department of Psychiatry and Mental Health, University of Cape Town, Cape Town, South Africa

**Keywords:** Acute stress disorder, Escitalopram, Posttraumatic stress disorder, Randomised controlled trial

## Abstract

**Background:**

A small literature suggests that pharmacotherapy may be useful in the prophylaxis of posttraumatic stress disorder in patients presenting with major trauma. There is relatively little data, however, on the use of selective serotonin reuptake inhibitors (SSRIs) in this context.

**Methods:**

24 week, double-blind placebo controlled study. 31 participants presenting immediately after trauma, and meeting diagnostic criteria for full or partial acute stress disorder were randomized to treatment with 10–20 mg of escitalopram or placebo daily for 24 weeks. 2 participants were excluded from the analysis due to early drop out, leaving 29 participants (escitalopram = 12, placebo = 17) for inclusion in an intent- to- treat analysis. Participants were followed up until 56 weeks, and assessed with the Clinician Administered PTSD Scale (CAPS). A mixed model repeated measures analysis of variance (RMANOVA) was undertaken to determine the efficacy of the intervention on the CAPS score.

**Results:**

There was a significant reduction in CAPS score over the course of treatment (F(7, 142) = 41. 58, p < 0.001) in both the escitalopram and placebo groups, with a greater reduction in CAPS score in the placebo group F(7, 142) = 2.12, p = 0.045. There were improvements on all secondary measures, including the Clinical Global Impressions scale, and scales assessing depression, anxiety and disability. Only functional disability outcomes (F(7, 141) = 2.13, p = .04), were significantly different between treatment and placebo groups. In the sample as a whole, improvement in scores were maintained at the 52 week follow-up. Side effects were comparable between the groups.

**Conclusions:**

These data are consistent with other recent work indicating that the SSRIs may not be efficacious in the prevention of PTSD. Nevertheless, the small sample size and baseline differences between groups limit the explanatory power of the study. Although a consideration of the possibility of medication prophylaxis in PTSD remains important, both from conceptual and clinical perspectives, caution is needed with regards to the use of SSRIs until their efficacy can be proven.

**Trial registration:**

Clinical Trials NCT00300313

## Background

Post-traumatic stress disorder (PTSD) is a chronic and debilitating condition that can have profound effects on functioning (work-place, family and social), and severely compromise quality of life. Community studies have suggested that at least 50% of adults will encounter a traumatic event in the course of their lifetime [1-3], while lifetime community prevalence rates of PTSD range from an average of 1.9% in Europe to 6.8% in the USA [4,5].

The psychological treatment with the best evidence for efficacy in treating and preventing PTSD is trauma-focused cognitive behaviour therapy (TF-CBT) [6-8]. As such clinical guidelines currently recommend TF-CBT as the treatment of choice for PTSD [9].

With regard to medication treatment, a number of randomized controlled trials (RCTs) have found that selective serotonin re-uptake inhibitors (SSRIs) are effective in reducing PTSD and its associated symptoms [10-13]. Furthermore, a systematic review of pharmacotherapy for PTSD [14] concluded that SSRIs are effective in treating PTSD in both the short and longer term. Although SSRIs are accepted first-line pharmacotherapy for acute and chronic PTSD [15,16], little is known about their use in the prevention of PTSD [17].

Two studies have addressed whether a SSRI, taken after exposure to a traumatic event, may reduce the risk for development of PTSD after an acute stress reaction. First, in an animal model of PTSD, injection of a SSRI, 1 hour after exposure to a traumatic event, reduced anxiety-like and avoidant behavior, decreased hyper-arousal response and significantly diminished the incidence of PTSD-like response [18]. The second study, an RCT of escitalopram for acute PTSD [19], found no positive effect of the drug over placebo or wait-list controls.

Further studies are needed to determine whether the early administration of PTSD prophylaxis, i.e. SSRIs, administered shortly after trauma exposure is efficacious for individuals with severe symptomatology. Further research is also needed to determine the optimal window for administration of medications used prophylactically [20].

## Objectives

The primary objectives of the study were to determine the efficacy of escitalopram in preventing PTSD in trauma-exposed participants with Acute Stress Disorder (ASD), and to determine its efficacy in reducing PTSD symptoms. The secondary objectives were to determine if escitalopram was efficacious in the prevention and improvement of depression and self-reported anxiety, depression and disability in trauma exposed participants with ASD.

## Methods

### Study design

This was a prospective, randomized, double-blind placebo controlled trial of escitalopram in the prevention of PTSD symptoms in traumatized adults with full or partial ASD.

### Sample

**Figure 1:**
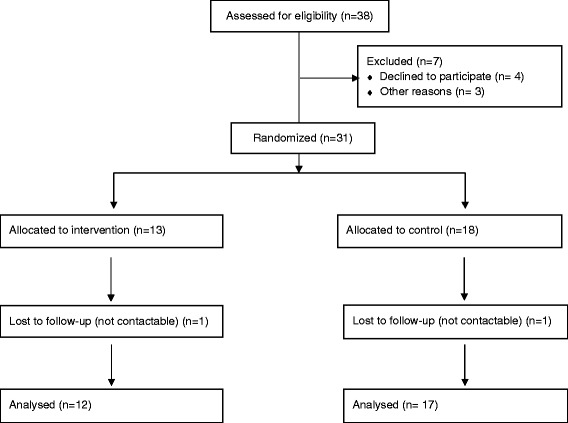
**Flow diagram of procedures.**

31 participants who met the following criteria were included in the study: (i) experience of a traumatic event, such as a vehicle collision or other accident, physical or sexual assault within the previous 4 weeks; (ii) between 18 and 65 years of age; (iii) sufficient knowledge of English in order to read, understand and sign the Informed Consent form as well as study procedures and assessment instruments; (iv) presence of either full DSM-IV criteria or intrusion and hyper-arousal criteria for ASD. Two participants (one from each treatment group) were excluded prior to the first post-baseline assessment and analysis as they were not contactable. The sample thus comprised of 12 participants on escitalopram and 17 on placebo (Figure 1).

Exclusion criteria comprised (i) refusal of any medication therapy; (ii) serious physical injury at inclusion (an Abbreviated Injury Scale (AIS) score of 3 or more); (iii) concomitant medications not allowed in the study (monoamine oxidase inhibitors [MAOIs], reversible inhibitors of monoamine oxidase A [RIMAs], mood stabilisers, antipsychotics or psychoactive herbal remedies within the 3 weeks prior to screening, anxiolytics or serotonergic agonists within the 2 weeks prior to screening, treatment with any anticonvulsant drug); lifetime DSM-IV-TR criteria for mania or bipolar disorder, schizophrenia, any personality disorder, mental retardation or pervasive developmental disorder, or cognitive disorder; significant suicide risk and/or a score of ≥5 on item 10 of the Montgomery Asberg Depression Rating Scale (MADRS) scale; history of severe suicide attempt; electroconvulsive therapy within the last year; currently serving in the South African security forces.; history of drug allergy or hypersensitivity to escitalopram or citalopram; illness severe enough to prevent participation in the study (including liver or renal insufficiency; cardiovascular, pulmonary, gastrointestinal, endocrine (including uncontrolled thyroid), neurological (including epilepsy), infectious, neoplastic, or metabolic disturbances; pregnant or breast-feeding; refusal of adequate contraceptive use (if female).

### Procedures

Approval for the study was obtained from the University of Cape Town, Faculty of Health Sciences Human Research Ethics Committee (HREC ref: 221/2006), as well as from hospitals and clinics where recruitment took place. The study was conducted in accordance with the Declaration of Helsinki, and informed consent was obtained from all participants.

Eligible participants were recruited from University of Cape Town (UCT) affiliated hospitals (Groote Schuur, G F Jooste, Gugulethu Day Hospital, Vanguard Community Health Clinic) and were randomly allocated to one of the two treatment groups (active or placebo) according to a computer-generated randomization list. Randomization numbers were assigned consecutively. Escitalopram (10 mg) and placebo were supplied as capsules of identical appearance. Medication was supplied in wallet cards that were dispensed at each visit. Wallet cards were identified by visit (i.e., visit 2, visit 3, etc.) with the escitalopram and placebo packed by the study pharmacist in sequentially numbered identical blister packs. Participants and investigators were blinded to treatment allocation and there were no instances of un-blinding.

Enrolled participants were dispensed wallet cards at each visit after visit 2 (baseline/ week 0) and instructed to take one capsule of 10 mg escitalopram, or placebo, which was titrated up to 20 mg per day at week 4, orally. A decrease in dose, back to one capsule of 10 mg, or placebo, was permitted for intolerable side effects. Participants were instructed to return the blister pack and all unused medication at their next visit. Investigators noted whether or not the pack was returned and if any medication remained in the participant’s file. Participants were also asked whether they had been compliant since the previous visit. Noncompliance resulted in that participant being withdrawn from the study.

Assessments were conducted every 2 weeks until visit 8 (week 12) and thereafter every 4 weeks. At each assessment, rating scales were administered and concomitant medication use and adverse events noted.

### Measures

-The essential features of ASD, and whether full or partial, were recorded according to DSM-IV criteria.

-Demographics details: sex, age, number of years of education, and, socioeconomic status.

-Trauma characteristics: type of event, event characteristics (evaluated by the PTSD Diagnostic Scale (PDS) [21].

-Physical condition: evaluated using the Abbreviated Injury Scale (AIS) [22].

-The following psychiatric rating scales were also used:

Primary measure:

Clinician Administered PTSD Scale (CAPS):

The CAPS [23] consists of 17 items that cover the core symptoms of PTSD according to the DSM-IV criteria. Eight additional items are included to measure the frequency and intensity of features frequently associated with PTSD. In addition, the CAPS includes five global rating scales that reflect the impact of symptoms on social and occupational functioning, general severity, any recent changes in severity, and the assessor’s evaluation of the validity of the participant’s report.

For each of the 17 items, the interviewer rates each of the frequency and the intensity of the symptoms on a 5 point scale rated from 0–4. A total score (ranges between 0–272) is calculated by summing the frequency and intensity scores for each item. A score of ≥50 indicates PTSD. The CAPS can also be scored according to the DSM diagnostic criteria for PTSD, rendering a dichotomous rating of the presence or absence of PTSD.

Secondary measures:

Clinical Global Impression Scales (CGI):

The CGI scales [24] consist of two separate scales: Clinical Global Impression – Severity (CGI-S) and Clinical Global Impression – Improvement (CGI-I). Both scales are rated from 1–7. The investigator uses his/her total clinical experience with this patient population to judge symptom severity at the time of rating (CGI-S). At follow-up overall improvement is rated (CGI-I).

Mini International Neuropsychiatric Interview (MINI 5.0.0):

The MINI [25] is a brief, structured diagnostic interview for clinical and research settings. It has a total of 120 questions that cover 17 current and past DSM-IV Axis I disorders.

Montgomery-Asberg Depression Rating Scale (MADRS):

The MADRS [26] is a depression rating scale consisting of 10 items that represent the core symptoms of depressive illness. The rating is based on a clinical interview with the participant, moving from broadly phrased questions about symptoms to more detailed ones, which allows a precise rating of severity, covering the last 7 days. The clinician who conducts the rating must decide whether the rating lies on the defined scale steps (0, 2, 4, 6) or between them (1, 3, 5), on a scale ranging from 0–60.

Visual Analogue Scale for Depression (VAS-D):

The VAS-D [27] is a self-rating scale, designed to assess depression severity, from a subjective point of view. The responder is required to indicate how depressed s/he is, on a scale ranging from 0–10.

Visual Analogue Scale for Anxiety (VAS-A):

The VAS-A [27] is a self-rating scale, designed to assess anxiety, from a subjective point of view. The responder is required to indicate how anxious s/he feels, on a scale ranging from 0–10.

Sheehan Disability Scale (SDS):

The SDS [28] is a 3-item self-rating scale of functional impairment. The items address the impact of symptoms of PTSD on three areas of functioning, namely, work, social, and family within the last 7 days.

### Data analyses

Data were captured for all visits where PTSD was assessed with the CAPS (weeks 0, 4, 12, 24, 32, 40, 48 and 56/ visits 2, 4, 8, 11, 13, 15, 17, 19). Demographic and baseline data were compared using chi-square/Fisher’s exact tests or analysis of variance (ANOVA). Assessment of PTSD prevention was based on the CAPS total score. The mean change from baseline (visit 2/week 0) to the week 24 endpoint on the CAPS was used as the primary outcome measure. Using an intent-to-treat sample, defined as all participants with at least one post-baseline CAPS assessment, efficacy for escitalopram vs. placebo was tested using a mixed model repeated measures analysis of variance (RMANOVA). The mixed model approach allows for patients with incomplete data to be included and utilizes the data that is available for all patients. Generalised Estimating Equation (GEE) models were used for categorical outcomes. Rates of PTSD were determined by the cut off of 50 points on the CAPS. Maintenance efficacy was determined by the change in CAPS scores between weeks 24 and 56. Improvement on secondary outcome measures were also evaluated. CGI-responders were defined as those with CGI-I scores of 2 (much improved) or 1 (very much improved) at week 24. All tests were two-tailed with p-values <0.05 considered significant. Frequencies of the most common adverse events are tabled and reported if present in more than one subject in either of the study groups.

## Results

The baseline demographic and clinical details of the 29 participants included in the study are listed in Tables 1 and 2. Trauma types did not differ between groups with the majority having experienced assault (physical or sexual assault: N = 20), and the remainder having been in a motor vehicle accident or been witness to a traumatic life event (N = 9).The only significant demographic difference between the escitalopram and placebo groups was with regards to ethnicity, as there were no coloured/ mixed race people in the escitalopram group. When we left people of colour (N = 6) out of the analyses, however, results were not much different. The placebo group had higher CAPS and MADRS scores and reported greater disability at baseline, with differences trending towards significance. On the MINI the most commonly reported disorders at baseline were current depression (N = 10) and PTSD (N = 14). Suicidality (N = 9), other anxiety disorders (N = 6), alcohol dependence or abuse (N = 5) and antisocial personality disorder (N = 1) were also reported.

**Table 1:** **Baseline demographic and descriptive details of participants**

**Total sample (N = 29)**	**Escitalopram (N = 12)**	**Placebo (N = 17)**	**Significance**
**Gender**			
Male: 19	N = 7	N = 12	
Female: 10	N = 5	N = 5	p = 0 .50
**Age** (mean ± SD in years)			
29.52 ± 8.17	31.33 ± 7.85	28.24 ± 8.38	p = 0.32
Ethnicity*			
Black African: 23	N = 12	N = 11	
Coloured/Mixed race: 6	N = 0	N = 6	p = 0.03
**Education** (mean ± SD in years)			
9.38 ± 2.27	9.25 ± 2.18	10.06 ± 2.82	p = 0.41
**Employment**			
Yes: 16	N = 6	N = 10	
No: 13	N = 6	N = 7	p = 0.64
**Annual income** (mean ± SD)			
$26190 ± 14483	30625 ± 14253	23462 ± 4489	p = 0.28
**AIS**			
Minor: 9	N = 5	N = 4	
Moderate: 11	N = 3	N = 8	
Serious: 8	N = 4	N = 4	p = 0.86
**Trauma types**			
Assault (physical/sexual): 20	N = 10	N = 10	
Other (mva/witnessing event): 9	N = 2	N = 7	p = 0.15

AIS: Acute Injury Scale; ASD: Acute Stress Disorder; mva: motor vehicle accident; SD: standard deviation.

*p < 0.05.

Note: numbers may not add up to 100% due to missing data.

**Table 2:** **Baseline clinical details of participants**

**Total sample (N = 29)**	**Escitalopram (N = 12)**	**Placebo (N = 17)**	**Significance**
**ASD**			
Full: 27	N = 12	N = 15	
Partial: 2	N = 0	N = 2	p = 0.39
**Any MINI disorder**			
Yes: 19	N = 7	N = 12	
No: 10	N = 5	N = 5	p = 0.50
**CAPS PTSD**			
Yes: 15	N = 4	N = 11	
No: 14	N = 8	N = 6	p = 0.09
**CAPS** (mean ± SD)			
55.10 ± 23.50	45.33 ± 21.43	62.00 ± 22.98	p = 0.06
**CGI-S** (mean ± SD)			
3.24 ± 0.87	3.08 ± 0.10	3.35 ± 0.79	p = 0.35
**MADRS** (mean ± SD)			
14.79 ± 9.56	11.92 ± 9.42	16.82 ± 9.40	p = 0.05
**VAS-D** (mean ± SD)			
4.45 ± 2.65	3.75 ± 2.60	4.94 ± 2.66	p = 0.24
**VAS-A** (mean ± SD)			
5.22 ± 2.16	5.33 ± 2.19	5.15 ± 2.21	p = 0.82
**SDS** (mean ± SD)			
12.10 ± 7.65	8.83 ± 6.46	14.41 ± 7.75	p = 0.05

CAPS: Clinician Administered PTSD Scale; CGI-S: Clinical Global Impressions Scale- severity; MADRS: MINI: MINI International Neuropsychiatric Interview; Montgomery Asberg Depression Rating Scale; SD: standard deviation; SDS: Sheehan Disability Scale; VAS-D: Visual Analogue Scale-Depression; VAS-A: Visual Analogue Scale- Anxiety.

Note: numbers may not add up to 100% due to missing data.

**Figure 2:**
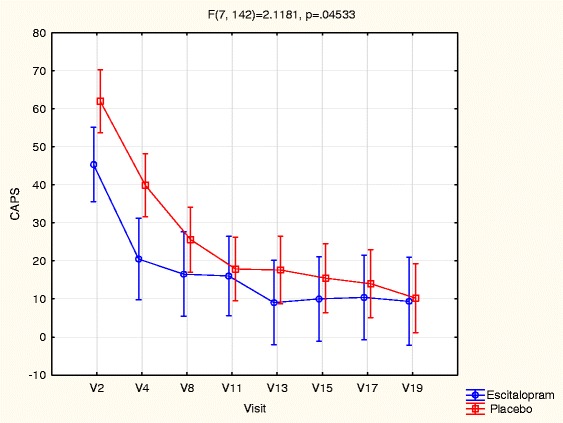
**Change in PTSD severity score across study time points.**

**Figure 3:**
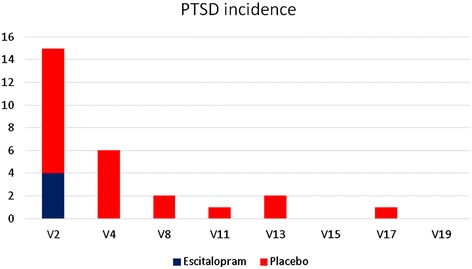
**Number of participants with PTSD at each study time point.**

There was a significant reduction in CAPS scores over the course of treatment (F(7, 142) = 41. 58, p ≤ 0.01) in both the escitalopram and placebo groups, in favour of the placebo group F(7, 142) = 2.12, p ≤ 0.01 (Figure 2).Thirty-three percent of participants in the escitalopram group met criteria for PTSD (CAPS score of 50 ≥) at baseline, all of whom were in remission by week 4 of treatment. This was maintained until the end of the follow-up period. Sixty-five percent of participants in the placebo group initially met criteria for PTSD. This number dropped to 6% at the end of treatment and was 6% at the end of the follow-up period (Figure 3). We were unable to perform GEE for PTSD incidence as the number of participants with PTSD was too low at the follow-up assessments.

CGI-S reduced significantly (F(7, 140) = 43.09, p ≤ 0.01), but there was no difference between treatment and placebo groups (F(7, 140) = 0.50, p = 0.83). On the CGI-I, 22 participants (75.9% of the sample) were classified as responders by the end of treatment, all of whom retained their responder status at 52-weeks. Based on the CGI-I, 9 subjects (75.0%) in the escitalopram group and 13 (76.5%) in the placebo group were “much improved” or “very much improved” at the end of the treatment phase.

There was a reduction of symptoms in all other impairment, depression and anxiety measures across the treatment period (p ≤ 0.01) which was maintained at the 52-week follow-up (Table 3). With the exception of functional impairment, as measured by the SDS, (F(7, 41) = 15.27, p = 0.04), there were also no significant differences (p > 0.05) between the escitalopram and placebo groups on symptom severity using secondary outcome measures. The number of participants meeting criteria for Any MINI diagnosis in each group also reduced across the trial, and almost reached significance (χ^2^ = 7.52 (3), p = 0.06), with similar reductions in both groups (χ^2^ = 0.30 (3), p = 0.96).

**Table 3:** **Change in scores across treatment and follow-up period (mean ± SE)**

	**Start (week 2) to end (week 24) of treatment**		**End of treatment (week 24) to 6 month follow-up (week 56)**	
	**Escitalopram**	**Placebo**	**Escitalopram**	**Placebo**
**CAPS**	−29.29 (±4.76)**	−44.11 (±3.81)**	−5.85 (±6.98)	−7.69 (±4.22)
**CGI-S**	−1.41 (±0.20)**	−1.50 (±0.16)**	−0.36 (±0.22)	−0.18 (±0.17)
**CGI-I**	−1.60 (±0.70)	−1.75 (±1. 70)	−1.00 (±0.00)	−1.42 (±0.52)
**MADRS**	−8.62 (±1.94)**	−11.99 (±1.55)**	−1.59 (±2.24)	−1.10 (±1.72)
**VAS-D**	−2.17 (±0.61)**	−2.89 (±0.49)**	−0.57 (±0.70)	−0.18 (±0. 45)
**VAS-A**	−2.81 (±0.72)**	−2.63 (±0.54)**	−0.32 (±0.77)	−1.01 (±0.60)
**SDS**	−5.58 (±1.58)**	−8.25 (±1.26)**	−1.96 (±1.82)	−1.16 (±1.40)

CAPS: Clinician Administered PTSD Scale; CGI-I: Clinical Global Impressions Scale- improvement; MADRS: Montgomery Asberg Depression Rating Scale; SDS: Sheehan Disability Scale; SE: Standar Error; VAS-D: Visual Analogue Scale- Depression; VAS-A: Visual Analogue Scale- Anxiety; *: p < 0.05; **: p < 0.01.

Adverse events are listed in Table 4. There were no significant group differences in the incidence of adverse events. The most commonly reported were stomach cramps/upset (n = 5), drowsiness (N = 4), sweating (N = 3) and increased appetite (N = 3). Due to side effects, dosage was reduced to 10 mg in 1 participant in the escitalopram group. Adherence to treatment was acceptable in both groups, with only 2 participants in each group withdrawn.

**Table 4:** **Number of patients with adverse events**

**Adverse event**	**Escitalopram**	**Placebo**
Increased appetite	2	1
Rash/itch	0	2
Drowsiness	2	2
Stomach upset/cramps	2	3
Sweating	2	1
Dizziness	1	1
Insomnia	1	1

## Discussion

The main finding of this study was that escitalopram was not more efficacious than placebo in reducing PTSD symptoms on the CAPS. There were also no differences in secondary outcomes of anxiety and depression. Functional impairment/disability scores, however, did reduce significantly more in the placebo group than in the escitalopram group. These findings are consistent with the work of Shalev and colleagues [19], who found that subjects on escitalopram did not experience greater improvement in symptoms than those who received placebo, and in fact fared worse at the 9-month assessment. Our results could, however, be an effect of regression to the mean given that the placebo group showed a trend towards significantly worse PTSD and depressive symptoms, as well as functional impairment, at baseline.

Several limitations should be noted. Most important is the group differences mentioned above, which limit the explanatory power of the study. A matching procedure would potentially have avoided these pre-existing differences between groups. The second is the relatively small sample size. Thirdly, subjects here are not necessarily representative of those with PTSD in the broader community, given that they were physically injured, hospitalized subjects, who were all highly symptomatic at inclusion. Fourth, participants were not matched on trauma type or ethnicity. Nonetheless, trauma type did not differ between groups and the exclusion of participants of mixed race from the analysis (as there were none in the escitalopram group) did not alter results.

## Conclusion

From a practical perspective, it seems premature to recommend the use of SSRIs in PTSD prophylaxis, given that both this study and that of Shalev and colleagues [19] did not find evidence of efficacy. Even so, given the small sample sizes in both studies, there is insufficient data to make a conclusive determination about the efficacy of SSRIs. Despite small randomized controlled trials of other pharmaco-prophylactic interventions (e.g. propranolol, hydrocortisone and gabapentin) not demonstrating unequivocal evidence of efficacy [20], with current investigations under way into the psychobiology of PTSD and the discovery of novel prophylactic targets, there is hope that efficacious preventive medications will be found [29].
